# Eosinopenia and neutrophil-to-lymphocyte count ratio as prognostic factors in exacerbation of COPD

**DOI:** 10.1038/s41598-021-84439-8

**Published:** 2021-02-26

**Authors:** Tomasz Karauda, Kamil Kornicki, Amer Jarri, Adam Antczak, Joanna Miłkowska-Dymanowska, Wojciech J. Piotrowski, Sebastian Majewski, Paweł Górski, Adam Jerzy Białas

**Affiliations:** 1 Department of Pneumology and Allergy, Medical University of Lodz, Lodz, Poland; 2 Department of General and Oncological Pulmonology, Medical University of Lodz, Lodz, Poland; 3 Department of Pathobiology of Respiratory Diseases, Medical University of Lodz, 22nd Kopcińskiego Street, 90-153 Lodz, Poland

**Keywords:** Diseases, Medical research, Risk factors

## Abstract

Exacerbations of Chronic Obstructive Pulmonary Disease (AECOPDs) are one of the most important clinical aspects of the disease, and when requiring hospital admission, they significantly contribute to mortality among COPD patients. Our aim was to assess the role of eosinopenia and neutrophil-to-lymphocyte count (NLR) as markers of in-hospital mortality and length of hospitalization (LoH) among patients with ECOPD requiring hospitalization. We included 275 patients. Eosinopenia was associated with in-hospital deaths only when coexisted with lymphocytopenia, with the specificity of 84.4% (95% CI 79.6–88.6%) and the sensitivity of 100% (95% CI 35.9–100%). Also, survivors presented longer LoH (P < 0.0001). NLR ≥ 13.2 predicted in-hospital death with the sensitivity of 100% (95% CI 35.9–100%) and specificity of 92.6% (95% CI 88.8–95.4%), however, comparison of LoH among survivors did not reach statistical significance (P = 0.05). Additionally, when we assessed the presence of coexistence of eosinopenia and lymphocytopenia first, and then apply NLR, sensitivity and specificity in prediction of in-hospital death was 100% (95% CI 35.9–100) and 93.7% (95% CI 90.1–96.3), respectively. Moreover, among survivors, the occurrence of such pattern was associated with significantly longer LoH: 11 (7–14) vs 7 (5–10) days (P = 0.01). The best profile of sensitivity and specificity in the prediction of in-hospital mortality in ECOPD can be obtained by combined analysis of coexistence of eosinopenia and lymphocytopenia with elevated NLR. The occurrence of a such pattern is also associated with significantly longer LoH among survivors.

## Introduction

Exacerbations of chronic obstructive pulmonary disease (AECOPDs) are one of the most important clinical aspects to the disease. The clinical manifestations of COPD exacerbations are highly variable and reflect broad heterogeneity in the pathobiology of the disease. Regardless of its variability, exacerbations are clearly associated with negative impact on the health status, increasing rates of hospitalization, readmission, and disease progression^1–3^. AECOPDs requiring hospital admission are the leading cause of hospitalization and significantly contribute to mortality among COPD patients^4^. Therefore, research on prognostic factors in this group is of special interest.

With a great deal of reasonable criticism, eosinopenia was already proposed as a marker of infection, in differentiating infectious from non-infectious causes of elevated C-reactive protein (CRP) and identifying sepsis or bacteremia^5^. Eosinopenia was also proposed to be a predictor of a short or long-term survival in some diseases, including AECOPD^6–10^. Thus, we reanalyzed the problem of eosinopenia in AECOPD patients. Additionally, we assessed another previously discussed, and logically linked with eosinopenia, prognostic factor—neutrophil-to-lymphocyte count (NLR). This parameter has also been studied as a marker of sepsis and predictor of bacteriemia^5^. The roles of both these parameters in AECOPD still remain ambiguous and controversial. The aim of this study was to assess the role of eosinopenia and NLR as markers of in-hospital mortality and length of hospitalization. The relationships between both parameters and their possible pathophysiological background were analyzed as well.

## Patients and methods

This study is a retrospective analysis of the data collected in the digital base of the Barlicki Memorial Teaching Hospital of the Medical University of Lodz. The protocol of the study was approved by the institutional ethics committee. Also, due to retrospective character of the study, in which consent would be impossible to obtain, concordantly with the Declaration of Helsinki, the Committee waived the need of such consent.

All methods in the study were performed in accordance with the relevant guidelines and regulations.

Enrollment of our study included only adult Caucasians with the exacerbation of COPD, diagnosed concordantly with current GOLD recommendations^3^. We included only exacerbations that required hospital admission, both of infectious and non-infectious character. Full blood count and white blood cell differentiation were examined in these patients, which was performed on the hospital admission. Venous blood was collected by venipuncture into tubes with ethylenediaminetetraacetic acid as an anticoagulant. The samples were examined with the automated hematology analyzer. Cell counting was performed using electrical impedance method. Arterial blood gases parameters were analyzed using automated analyzer.

The exclusion criteria were any other known chronic lung disease, any hematological disorder—active, or in the past medical history, any active malignancy, exacerbation of respiratory symptoms associated with other acute causes. We also excluded patients with clinically and radiologically proven pneumonia and those, who received any dose of systemic corticosteroids prior to the admission. Additionally, we excluded all patients who were transferred to the hospital by an ambulance, because retrospective character of the study would preclude reliable verification that these patients did not receive corticosteroids prior to the hospital admission.

AECOPD was defined as an event characterized by a rapid decline in the patient’s respiratory symptoms that is beyond normal day-to-day variations and lead to changes in medication. In-hospital mortality was defined as any AECOPD-related death after the hospital admission.

NLR was calculated as a ratio of absolute counts of peripheral blood neutrophils to lymphocytes.

Length of hospitalization was assessed among survivors to avoid false shortage of hospitalization time in case of early in-hospital death.

Continuous data was presented as the mean with SD or median with interquartile range (IQR), depending on the distribution of data. In comparing multiple groups, one-way ANOVA with pairwise comparisons using t tests with pooled SD or Kruskal–Wallis rank sum test with pairwise comparisons using Mann–Whitney U test was used according to tests assumptions. Bonferroni method was used for P value adjustment. Receiver operating characteristics (ROC) with area under the ROC curve (AUROC) analyses were preformed to measure the accuracy of absolute eosinophil count and NLR in prediction of in-hospital mortality and to identify its cut-off values for further analysis. Variables were compared using the unpaired Student’s t-test, Welch t-test or the Wilcoxon rank sum test with continuity correction, depending on data normality and homogeneity of variance. Categorical data were presented as absolute value and percentage. Such data were compared using Chi-square test or Fisher’s exact test according to test assumptions. Correlation analysis was performed using Kendall's rank correlation tau. Statistical analysis was performed using R software^11^.

## Results

### General characteristics

We included 275 patients. Five patients (1.82%) died in hospital. Baseline population data are presented in Table 1. Majority of patients suffered from infectious AECOPD (n = 146; 53.09%). This group did not differ significantly in eosinophil count from non-infectious AECOPD [0.07 (0.02–0.18) vs 0.1 (0.03–0.21); *P* = 0.09]. However, in infectious AECOPD we observed significantly higher NLR values [4.3 (1.89–9.27) vs 2.6 (1–4.93); *P* = 0.0003].

**Table 1:** Baseline study data.

Parameter	Total n = 275
Age (years), mean (SD)	69.42 (9.59)
Male, n (%)	152 (55.27)
Active smoker, n (%)	102 (37.09)
**Character of ECOPD**	
Infectious, n (%)	146 (53.09)
CRP (mg/l), median (IQR)	10.88 (3.4–25.26)
WBC (× 10^3^/μl), median (IQR)	9.49 (7.77–12.24)
Eosinophils (× 10^3^/μl), median (IQR)	0.09 (0.02–0.19)
Lymphocytes (× 10^3^/μl), median (IQR)	1.6 (1–2.15)
Neutrophils (× 10^3^/μl), median (IQR)	6.7 (4.85–9.9)
NLR, median (IQR)	3.19 (1.48–6.96)
Hemoglobin (g/dl), median (IQR)	14 (12.8–15.15)
pH, median (IQR)	7.42 (7.39–7.45)
pCO_2_ (mmHg), median (IQR)	38.7 (35.25–45.28)
pO_2_ (mmHg), median (IQR)	63 (55.4–72)
SaO_2_ (%), median (IQR)	92.4 (89–94.88)
HCO^3−^ (mEq/l), median (IQR)	25.3 (23.45–28.1)
BE (mEq/l), median (IQR)	1 (− 0.63 to 2.93)
**Comorbidities**	
Ischemic heart disease, n (%)	86 (31.27)
AF^a^, n (%)	31 (11.27)
Arrhythmia other than AF^b^, n (%)	8 (2.91)
Congestive heart failure, n (%)	116 (42.18)
Arterial hypertension, n (%)	179 (65.09)
Type 2 diabetes, n (%)	66 (24)
Others, n (%)	152 (55.27)

*AF* atrial fibrillation, *CRP* c-reactive protein, *IQR* interquartile range, *WBC* white blood count.

^a^First detected in the admission, paroxysmal, persistent or permanent.

^b^In the admission or in the past medical history.

**Figure 1:**
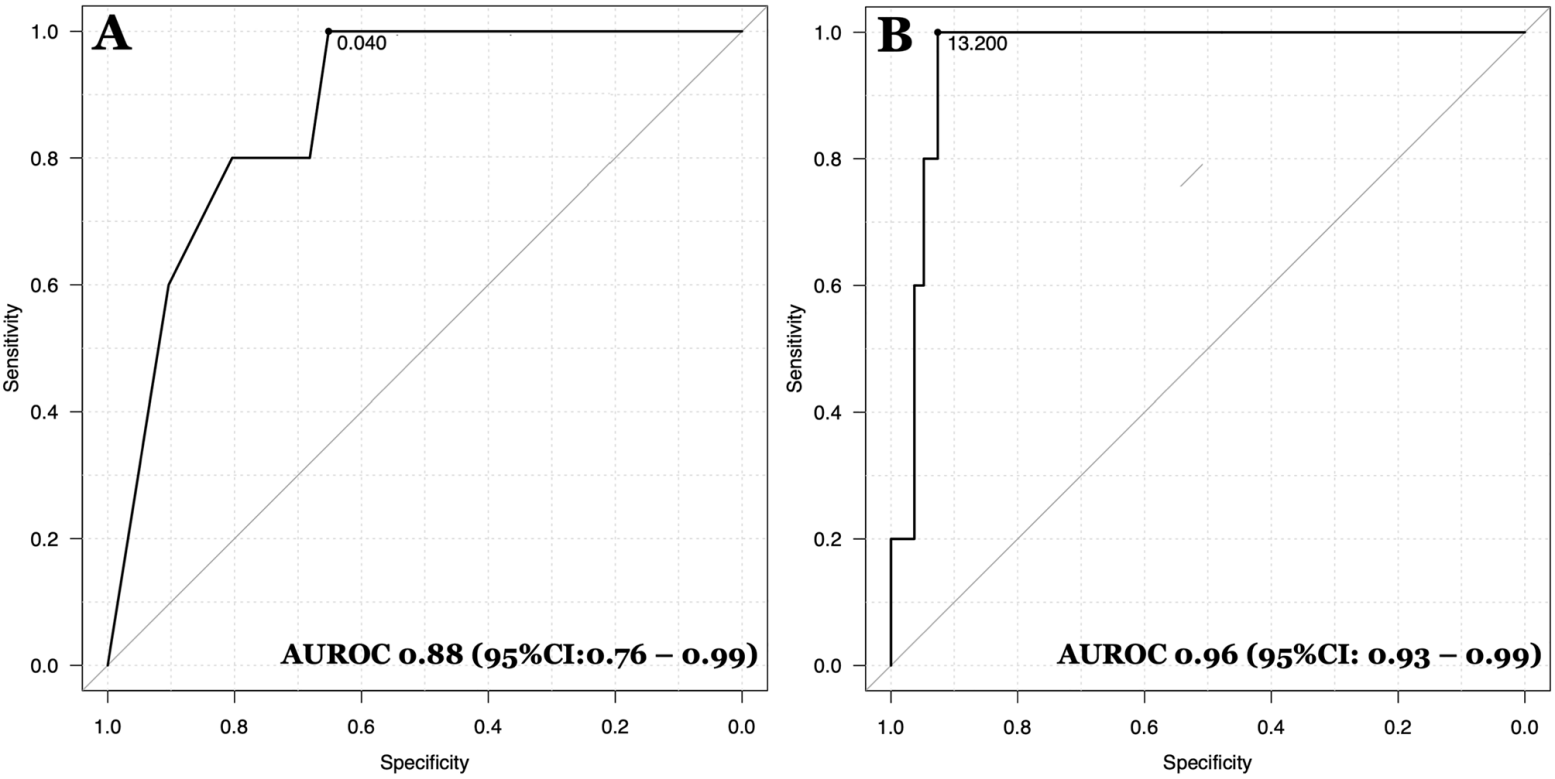
The ROC curve for absolute eosinophil count (**A**) and NLR (**B**) as predictors of in-hospital mortality. Cut-off points were marked with specificity and sensitivity values in brackets.

The ROC curves for absolute counts of eosinophils and NLR in peripheral blood in prediction of in-hospital death are presented in Fig. 1. The AUROC for eosinophils was 0.88 (95% CI 0.76–0.99) and for NLR was 0.96 (95% CI 0.93–0.99).

We also analyzed AUROC for the components of NLR, however its values were lower—0.92 for lymphocyte count, identifying 1 × 10^3^/μl as a cut-off value and 0.77 for neutrophil count with cut off value of 7.3 × 10^3^/μl.

### Eosinopenia

In ROC analysis, an absolute number of eosinophils ≤ 0.04 × 103/µl was identified as an optimal cut-off point for eosinopenia. Using this cut-off value, eosinopenia was diagnosed in 99 (36%) patients. All five patients who died presented on hospital admission with eosinopenia (*P* = 0.006 for the Fisher's Exact Test). This value was characterized by sensitivity of 100% (95% CI 35.9–100%), however specificity of 65.2% (95% CI 59.2–70.9%). The diagnostic accuracy was 0.66 (95% CI 0.6–0.71).

Comparison of study parameters upon stratification by presence of EP is presented in Table 2.

**Table 2:** Study parameters upon stratification by eosinophil count.

Parameter	Eosinopenia n = 99 (36%)	Normal eosinophil count n = 176 (64%)	P-value
Age (years), mean (SD)	70.24 (9.48)	68.96 (9.64)	0.29
Male, n (%)	51 (51.51)	101 (57.39)	0.42
Active smoker, n (%)	58 (58.59)	44 (25)	< 0.0001
Length of hospitalization^c^ (days), median (IQR)	8 (6–12.75)	7 (5–10)	0.004
**Character of ECOPD**			
Infectious, n (%)	60 (60.61)	86 (48.86)	0.06
CRP (mg/l), median (IQR)	15.53 (6.79–30.29)	10.14 (2.79–23.41)	0.07
WBC (× 10^3^/μl), median (IQR)	11.4 (8.17–13.2)	8.9 (7.67–11.57)	0.0007
Lymphocytes (× 10^3^/μl), median (IQR)	1.1 (0.7–1.8)	1.8 (1.2–2.4)	< 0.0001
Neutrophils (× 10^3^/μl), median (IQR)	9.2 (6.1–11.55)	6.1 (4.6–7.95)	< 0.0001
NLR, median (IQR)	8.44 (4.37–12.72)	2.26 (0.14–3.69)	< 0.0001
Hemoglobin (g/dl), median (IQR)	13.9 (12.6–15.15)	14.2 (13–15.13)	0.29
pH, median (IQR)	7.42 (7.39–7.46)	7.42 (7.39–7.45)	0.65
pCO_2_ (mmHg), median (IQR)	38.9 (35.2–47.65)	38.7 (35.3–44.5)	0.86
pO_2_ (mmHg), median (IQR)	63.9 (54.5–73.5)	63 (55.65–71)	0.96
SaO_2_ (%), median (IQR)	92.7 (88.05–95.3)	92.4 (89.65–94.8)	0.86
HCO^3−^ (mEq/l), median (IQR)	26 (23.93–28.68)	24.95 (23.2–27.98)	0.1
BE (mEq/l), median (IQR)	1.5 (− 0.08 to 3.73)	0.75 (− 0.7 to 2.48)	0.04
**Comorbidities**			
Ischemic heart disease, n (%)	37 (37.37)	49 (27.84)	0.1
AF^a^, n (%)	20 (20.2)	11 (6.25)	0.0004
Arrhythmia other than AF^b^, n (%)	2 (2.02)	6 (3.41)	0.72
Congestive heart failure, n (%)	45 (45.45)	71 (40.34)	0.41
Arterial hypertension, n (%)	57 (57.58)	122 (69.32)	0.05
Type 2 diabetes, n (%)	26 (26.26)	40 (22.73)	0.51
Others, n (%)	83 (83.84)	69 (39.2)	0.0003

*AF* atrial fibrillation, *CRP* c-reactive protein, *IQR* interquartile range, *WBC* white blood count.

^a^First detected in the admission, paroxysmal, persistent or permanent.

^b^In the admission or in the past medical history.

^c^Among survivors.

We found a significant negative correlation between eosinophil count and length of hospitalization (τ = − 0.11; *P* = 0.008), CRP (τ = − 0.11; *P* = 0.02); WBC (τ = − 0.15; *P* = 0.0004) and BE (τ = − 0.1; *P* = 0.03).

### NLR

Elevated NLR was identified when the value was ≥ 13.2. Such values were observed among 25 (9.09%) patients, including all five patients who died during hospitalization. This cut-off point was characterized by sensitivity of 100% (95% CI 35.9–100%) and specificity estimated for 92.6% (95% CI 88.8–95.4%). The diagnostic accuracy was 0.93 (95% CI 0.9–0.96). Comparison of study parameters upon stratification by NLR value is presented in Table 3.

**Table 3:** Study parameters upon stratification by NLR value.

Parameter	NLR ≥ 13.2 n = 25 (9.09%)	NLR < 13.2 n = 250 (90.91%)	P-value
Age (years), mean (SD)	70.24 (10.83)	69.34 (9.47)	0.66
Male, n (%)	14 (56)	138 (55.2)	0.94
Active smoker, n (%)	13 (52)	89 (35.6)	0.11
Length of hospitalization^c^ (days), median (IQR)	10 (7–12.5)	7 (5–10)	0.05
**Character of ECOPD**			
Infectious, n (%)	17 (68)	129 (51.6)	0.12
CRP (mg/l), median (IQR)	17.34 (8.92–22.6)	10.73 (3.37–25.31)	0.5
WBC (× 10^3^/μl), median (IQR)	11.9 (10.86–16)	9.13 (7.68–12)	< 0.0001
Eosinophils (× 10^3^/μl), median (IQR)	0.01 (0–0.02)	0.1 (0.03–0.21)	< 0.0001
Hemoglobin (g/dl), median (IQR)	14.1 (13–14.9)	14 (12.8–15.18)	0.83
pH, median (IQR)	7.42 (7.37–7.43)	7.42 (7.39–7.45)	0.12
pCO_2_ (mmHg), median (IQR)	42.3 (35.8–52.4)	38.7 (35–44.5)	0.2
pO_2_ (mmHg), median (IQR)	59.3 (54–65.1)	63.3 (55.4–73.1)	0.08
SaO_2_ (%), median (IQR)	90 (86–92.7)	92.7 (89.4–95.1)	0.008
HCO^3−^ (mEq/l), median (IQR)	26.75 (24.03–29.55)	25.2 (23.35–28.1)	0.31
BE (mEq/l), median (IQR)	1.6 (− 1.18 to 4.5)	1 (− 0.6 to 2.9)	0.78
**Comorbidities**			
Ischemic heart disease, n (%)	9 (36)	77 (30.8)	0.59
AF^a^, n (%)	6 (24)	25 (10)	0.05
Arrhythmia other than AF^b^, n (%)	0 (0)	8 (3.2)	1.0
Congestive heart failure, n (%)	14 (56)	102 (40.8)	0.14
Arterial hypertension, n (%)	13 (52)	166 (66.4)	0.15
Type 2 diabetes, n (%)	4 (16)	62 (24.8)	0.33
Others, n (%)	15 (60)	137 (54.8)	0.62

*AF* atrial fibrillation, *CRP* c-reactive protein, *IQR* interquartile range, *WBC* white blood count.

^a^First detected in the admission, paroxysmal, persistent or permanent.

^b^In the admission or in the past medical history.

^c^Among survivors.

Kendall's test did not detect significant correlation between SaO_2_ and NLR (τ = − 0.06; *P* = 0.13).

In Table 2 we can see that patients who presented with eosinopenia had significantly higher NLR values: 8.44 (4.37–12.72) vs 2.26 (0.14–3.69); *P* < 0.0001. We observed parallel situation when we analyzed results from Table 3 – patients with high NLR values had significantly lower eosinophil count: 0.01 (0–0.02) × 10^3^/μl vs 0.1 (0.03–0.21) × 10^3^/μl; *P* < 0.0001.

Kendall's rank correlation tau revealed significant correlation between eosinophil and lymphocyte count: τ = 0.28 (*P* < 0.0001) and negative correlation with NLR: τ = − 0.38 (*P* < 0.0001).

### Coexistence of eosinopenia with lymphocytopenia

The coexistence of eosinopenia with lymphocytopenia (defined as lymphocyte count ≤ 1 × 10^3^/μl) was observed in 47 (17.09%) patients and only such pattern was associated with in-hospital deaths in the analyzed group. There were no deaths in patients who presented with eosinopenia without lymphocytopenia (*P* = 0.0001 for the Fisher's Exact Test). The coexistence of eosinopenia with lymphocytopenia was characterized by higher specificity than previously analyzed eosinopenia [84.4% (95% CI 79.6–88.6%) vs 65.2% (95% CI 59.2–70.9%) and the same sensitivity of 100% (95% CI 35.9–100%). The diagnostic accuracy was 0.84 (95% CI 0.8–0.89).

**Figure 2:**
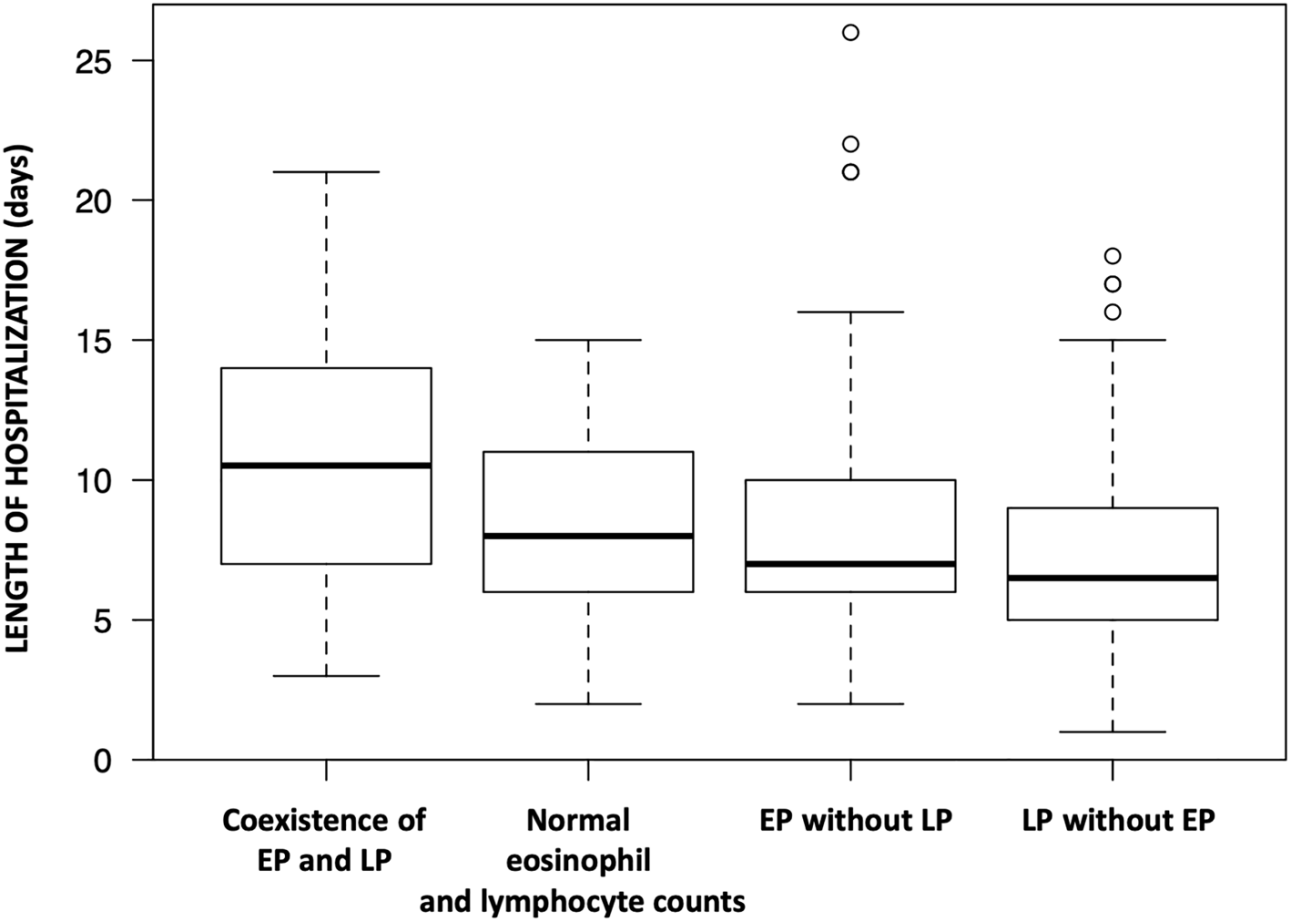
Boxplot for the comparison of length of hospitalization upon stratification by the presence of eosinopenia and lymphocytopenia coexistence. *EP* eosinopenia, *LP* lymphocytopenia.

Taking into account above findings, we reanalyzed the eosinopenia group in the context of previously analyzed study parameters. Results of additional analyses are presented in Table 4, which illustrates study parameters upon stratification by coexistence of eosinopenia and lymphocytopenia. Kruskal Wallis rank sum test, after pairwise comparisons, showed that only coexistence of eosinopenia with lymphocytopenia was associated with longer hospitalization (*P* = 0.006 vs normal eosinophils and lymphocytes; *P* = 0.02 for eosinopenia alone, and *P* = 0.00004 vs lymphocytopenia alone). Length of hospitalization in isolated eosinopenia or lymphocytopenia subgroups was not significantly different than in patients with normal counts of these cells (*P* = 1.0 and *P* = 0.12 respectively)—Fig. 2.

**Table 4:** Study parameters upon stratification by coexistence of eosinopenia and lymphocytopenia.

Parameter	Normal eosinophil and lymphocyte count n = 90 (32.73%)	Lymphocytopenia without eosinopenia n = 86 (31.27%)	Eosinopenia without lymphocytopenia n = 52 (18.91%)	Eosinopenia with lymphocytopenia n = 47 (17.09%)	*P*-value
Age (years), mean (SD)	68.92 (10.11)	69 (9.19)	69.33 (8.85)	71.26 (10.12)	0.55
Male, n (%)	40 (44.44)	61 (70.93)	26 (50)	25 (53.19)	0.004
Active smoker, n (%)	25 (27.78)	19 (22.09)	34 (65.38)	24 (51.06)	< 0.0001
Length of hospitalization^c^ (days), median (IQR)	8 (6–11)	6.5 (5–9)	7 (6–10)	10.5 (7–14)	< 0.0001
**Character of ECOPD**					
Infective, n (%)	39 (43.33)	47 (54.65)	29 (55.77)	31 (65.96)	0.08
CRP (mg/l), median (IQR)	9.62 (2.78–19.74)	10.71 (3.29–29.96)	18.62 (7.57–29.3)	13.52 (6.61–29)	0.22
WBC (× 10^3^/μl), median (IQR)	9.72 (8.29–11.97)	8.34 (6.9–9.83)	11.6 (8.58–13.2)	10.86 (8.04–13.54)	< 0.0001
Eosinophils (× 10^3^/μl), median (IQR)	0.18 (0.1–0.28)	0.14 (0.08–0.27)	0.02 (0.01–0.03)	0.01 (0–0.02)	< 0.0001
Lymphocytes (× 10^3^/μl), median (IQR)	2.4 (2–2.8)	1.2 (0.9–1.5)	1.75 (1.4–2.23)	0.7 (0.6–0.9)	< 0.0001
Neutrophils (× 10^3^/μl), median (IQR)	6 (4.7–8.18)	6.2 (4.5–7.5)	8.05 (6.65–11.4)	10 (6.95–12.45)	< 0.0001
NLR, median (IQR)	2.15 (1.03–3.09)	2.62 (0.09–5.68)	4.65 (2.34–7.09)	12.78 (9.88–18.04)	< 0.0001
Hemoglobin (g/dl), median (IQR)	14.5 (13.3–15.2)	13.7 (12.83–15)	13.85 (12.5–15.13)	14 (12.6–15.25)	0.33
pH, median (IQR)	7.43 (7.39–7.46)	7.42 (7.39–7.44)	7.44 (7.4–7.46)	7.42 (7.37–7.43)	0.06
pCO_2_ (mmHg), median (IQR)	37.9 (35–42.6)	39.75 (36.53–46.3)	37.8 (34.55–41.13)	41.3 (35.75–55.35)	0.06
pO_2_ (mmHg), median (IQR)	64.9 (57–75.2)	61 (55.1–68.13)	64 (56.43–74.63)	60.2 (52.45–72.5)	0.33
SaO_2_ (%), median (IQR)	92.9 (90.1–95.3)	91.8 (88.73–93.8)	93.85 (90.3–95.85)	91.6 (86.6–94.8)	0.07
HCO^3−^ (mEq/l), median (IQR)	24.8 (22.9–27.18)	25.1 (23.2–28.1)	25.3 (23.6–27.7)	26.9 (24.3–29.8)	0.08
BE (mEq/l), median (IQR)	0.75 (− 0.78 to 2.2)	0.75 (− 0.7 to 2.83)	1.2 (− 0.1 to 3)	1.7 (0–4.3)	0.18
**Comorbidities, n (%):**					
Ischemic heart disease	23 (25.56)	26 (30.23)	19 (36.54)	18 (38.3)	0.37
AF^a^	4 (4.44)	7 (8.14)	12 (23.08)	8 (17.02)	0.003
Arrhythmia other than AF^b^	3 (3.33)	3 (3.49)	1 (1.92)	1 (2.13)	1.0
Congestive heart failure	38 (42.22)	33 (38.37)	19 (36.54)	26 (55.32)	0.22
Arterial hypertension	62 (68.89)	60 (69.77)	34 (65.38)	23 (48.94)	0.08
Type 2 diabetes	22 (24.44)	18 (20.93)	20 (38.46)	6 (12.77)	0.02
Others	36 (40)	47 (54.65)	42 (80.77)	27 (57.45)	< 0.001

*AF* atrial fibrillation, *CRP* c-reactive protein, *IQR* interquartile range, *WBC* white blood count.

^a^First detected in the admission, paroxysmal, persistent or permanent.

^b^In the admission or in the past medical history.

^c^Among survivors.

When we first assess the presence of coexistence of eosinopenia and lymphocytopenia, and then apply NLR, we can achieve the best sensitivity and specificity compared to the situation when these parameters are used individually: 100% (95% CI 35.9–100) and 93.7% (95% CI 90.1–96.3), respectively. The diagnostic accuracy for such approach was 0.94 (95% CI 0.90–0.96).

Also, among survivors, occurrence of such pattern was associated with significantly longer hospital stay: 11 (7–14) vs 7 (5–10) days, P = 0.01.

## Discussion

Our aim was to assess the role of eosinopenia, lymphocytopenia and NLR as markers of in-hospital mortality and length of hospitalization (LoH) among patients with AECOPD requiring hospitalization.

Such approach seems to be justified by pathophysiological background. Namely, we would analyze eosinopenia in the context of considering AECOPD as a state of an acute stress. Undoubtedly, COPD is associated with both physical and emotional stress. Extremal, but pictorial evidence for possible levels of stress which can be released in this condition may be presented in context of relatively frequent association between COPD and Tako-Tsubo cardiomyopathy, which is linked with a rapid elevation of circulating catecholamine, triggered by emotional and/or physical stress, as a key mechanism^12^. Also, Zurfluh et al. reported a time-dependent effect with higher levels pointing towards higher mortality at short term associated with activation of stress hormones (particularly cortisol and cortisone)^13^.

We should also remember that acute stress, and associated role of adrenal glucocorticoids in this process, is not the only cause of eosinopenia in peripheral blood. There are other contributing mechanisms including migration of eosinophils to the site of inflammation, rapid peripheral sequestration, suppression of egress of mature eosinophils from the bone marrow, and suppression of eosinophils production^14,15^.

Eosinopenia in AECOPD was concluded to have some clinical implications in already published studies. Namely, Holland et al.^6^ reported that patients with eosinopenia had a longer hospital stay and were more likely to die in hospital. Additionally, Rahimi-Rad et al. found an association between eosinopenia and an unfavorable prognosis within 30-day after discharge^7^. Both Holland et al. and Rahimi-Rad et al. used cut-off value of 40 cells/mm^3^ to diagnose eosinopenia. In the past, we also reported that eosinopenia would be a prognostic factor of in-hospital mortality in ECOPD^16^, however, in the present study, we used the analysis which was not applied by above mentioned authors, therefore ROC curve assessment. From one hand, AUROC analysis may still justify such statement, but from the other hand, relatively low specificity 65.2% of this marker decreased our enthusiasm significantly.

Also, for further evaluation of the clinical implications of eosinopenia, discussion of its eventual consequences should be taken into account. First, we should start with the physiological role of these cells in the immune response. Among others, eosinophils promote humoral immunity by priming of B cells and have a role in the maintenance of type-2 immunity^17^, and regulation of T-helper-1 and Th2 immunity^18^. However, paradoxical to their physiological effects, evidence suggests that a reduction of eosinophils appears to have no negative effect on normal health. This statement is based not only on animal studies, but also on humans who received monoclonal antibodies reducing eosinophil count^19^. Therefore, even if we conclude that we indirectly assessed the exacerbation of stress or other contributing mechanisms, the occurrence of eosinopenia itself probably has no further significant clinical importance.

Taking into account all above mentioned, and the fact that glucocorticoids can reduce eosinophils, raise neutrophils and reduce lymphocytes^20,21^ we decided to evaluate eosinopenia in a broader context, by adding the assessment of lymphocytopenia and NLR into analysis. The relation between NLR and in-hospital mortality also was previously reported in patients with AECOPD^22^. Yao et al. presented ROC curve analysis for using NLR to predict in-hospital mortality—AUROC was 0.803, and an optimal cut-off value was 6.24. Authors reported the sensitivity of 81.08%, and specificity of 69.17%. We observed both better sensitivity and specificity for NLR, however we obtained higher cut-off value in ROC curve analysis. In our study, NLR presented much better accuracy parameters and higher value of AUROC than obtained for eosinopenia. Moreover, we have observed that these parameters are not fully independent and that there is a significant relationship between the values of eosinophil count and NLR. That is why we started to analyze these associations in the context of individual components of NLR, observing intriguing interplay between eosinophils and lymphocytes. Interestingly, patients who presented with the coexistence of eosinopenia and lymphocytopenia had the lowest counts of both eosinophils and lymphocytes. This is justified because these patients probably had the highest level of stress, which can suppress both types of cells. However, this relationship itself seems to also have interesting clinical implications.

In turn, eosinophils and neutrophils are granulocytes that originate from the same myeloblast progenitor in the bone marrow, and upon differentiating, each of these granulocytes leaves the marrow and migrates to the inflamed tissues to enact their effector functions. From the other hand, lymphocytes, which originate from the different progenitor, secrete cytokines that coordinate eosinophil and neutrophil responses. From one hand, type 2 CD4^+^ T cells and type 2 innate lymphoid cells produce IL-5 that prompts eosinophil production^23,24^, while from the other hand, Th17 cells, CD8^+^ T cells, γδ T cells, and type 3 innate lymphoid cells secrete IL-17A, leading to neutrophil maturation^25,26^. Infection by different types of pathogens may lead to different effects—either eosinophil, or neutrophil accumulation^27^. Wiesner et al. reported that Rag2/IL-2Rγ^−/−^ mice that lack lymphocytes, and observed that without lymphocytes, infected mice had significantly impaired eosinophilia compared with similarly infected wild-type mice, yet neutrophil accumulation remained unimpaired. Authors also concluded that singular elimination of Th cells (while leaving all other lymphocyte subsets intact, including ILCs and NK cells) may result in a loss of eosinophils that can be replaced by IL-17A-dependent neutrophilia^27^.

Focusing on COPD population, Freeman et al. observed decreased concentration of CD4^+^ and CD8^+^ T cells in peripheral blood during AECOPD. From the other hand, Ross et al. observed that exacerbated airway neutrophilia in cigarette smoke–exposed mice infected with nontypeable *Haemophilus influenzae*, large subgroup of bacterial AECOPD, was associated with an induction of IL-17A^28^. There is also evidence for a decrease in both total CD4+ and CD8^+^ T cell counts after infusion of cortisol. The effect started 90 min after infusion^29^.

How would these issues contribute to an increase in mortality? Namely, CD4^+^ T cells are major players involved in responses to infectious diseases, enabling B cells to differentiate into plasma cells, helping CD8^+^ T cells develop into cytotoxic cells, as well as are required for long-term CD8 memory generation. CD4^+^ T cells also mediate activation of macrophages, playing a critical role in the viral and bacterial control^30–35^. Therefore, decrease in this population of cells would result in catastrophic effects for homeostasis of the immune response.

The above-mentioned evidence may partially and indirectly justify our observations and allow to draw a hypothetical pathway that lymphocytopenia would be a primary pathology, which leads to eosinopenia and neutrophilia. Playing together with stress response, which potentially aggravates such pattern, these factors may contribute to a significantly increased risk of in-hospital death. However, for confirmation of these hypotheses, there is a need for a prospective study which directly will analyze all above mentioned relationships: stress response, lymphocytes subpopulations assessment, eosinophil and neutrophil counts with broad profile of cytokines, including IL-17A.

There are some major limitations of our study, which need to be considered. As a retrospective study, it is burdened by all limitations associated with this type of data collection, including an absence of data regarding potential confounding factors. As the most important we indicate above mentioned lack of measurement of stress hormones and inflammatory cytokines. It should be also worth to diagnose pathogens responsible for infectious AECOPDs. Taking into account the above-mentioned issues, the results of our study should be considered as hypothesis generating and should be furtherly confirmed in a sufficiently powered prospective analysis, designed taking into account above mentioned limitations.

## Conclusions

All analyzed parameters in our study have certain value as prognostic factors of in-hospital mortality in AECOPD. However, the best profile of sensitivity and specificity can be obtained by combined analysis of coexistence of eosinopenia and lymphocytopenia with elevated NLR. Occurrence of such pattern is also associated with significantly longer time of hospitalization among survivors.
